# Using environmental DNA methods to survey for rare groundwater fauna: Detection of an endangered endemic cave crayfish in northern Alabama

**DOI:** 10.1371/journal.pone.0242741

**Published:** 2020-12-10

**Authors:** Spencer H. Boyd, K. Denise Kendall Niemiller, Katherine E. Dooley, Jennifer Nix, Matthew L. Niemiller

**Affiliations:** Department of Biological Sciences, The University of Alabama in Huntsville, Huntsville, AL, United States of America; University of Hyogo, JAPAN

## Abstract

The conservation and management of subterranean biodiversity is hindered by a lack of knowledge on the true distributions for many species, e.g., the Wallacean shortfall. In recent years, several studies have demonstrated the potential of environmental DNA (eDNA) as an effective approach to detect and monitor biodiversity, including rare, threatened, and endangered taxa. However, there are few eDNA studies of groundwater fauna. Here we report the results of the development and implementation of an eDNA assay targeting a short fragment of the mitochondrial CO1 locus of a critically imperiled cave crayfish, the Sweet Home Alabama Cave Crayfish (*Cambarus speleocoopi*), known from just four cave systems in the Interior Plateau karst region of northern Alabama. We detected *C*. *speleocoopi* DNA from water samples collected at 5 of 16 sites sampled (caves and springs), including two historical sites as well as three additional and potentially new sites in Marshall County, Alabama. All three of these sites were within 2 km of historical sites. Our study is the first to detect a groundwater crustacean in the Interior Plateau karst region. Additionally, our study contributes to the growing literature that eDNA is a viable complementary tool for detection and monitoring of a fauna that is difficult to survey and study using traditional approaches.

## Introduction

Effective conservation and management of biodiversity is limited by a lack of knowledge on the distributions of species. This biodiversity knowledge gap known as the Wallacean shortfall [1] is particularly prominent for fauna that live in groundwater and other subterranean ecosystems [2, 3]. This is, in part, because subterranean habitats are extremely challenging to access and survey using traditional approaches, such as visual surveys and trapping (i.e., the Racovitzan shortfall; [4]). Most stygofauna—obligate groundwater species—are thought to have small, restricted distributions (i.e., short-range endemics; *sensu* [5]) and limited dispersal ability [6] and, consequently, are of high conservation concern. Thus, the development of sound management strategies and measurable conservation priorities for most stygofauna is exceedingly difficult.

An increasingly popular complement, or in some cases alternative, to traditional sampling and monitoring approaches for many aquatic species is environmental DNA (eDNA) that leverages DNA shed by organisms into their surrounding habitats. eDNA represents a powerful new tool for ecologists and conservation biologists to detect and monitor biodiversity rapidly, nondestructively, and potentially cost-effectively [7–10]. This approach has been successfully employed in many freshwater habitats, including rivers, streams, lakes, and ponds, and applied to an assortment of vertebrate and invertebrate taxa [9–12; and references therein], including several taxa considered rare, threatened or endangered [e.g., 13–16]. Few studies have employed eDNA for the detection and monitoring of cave and groundwater macrofauna, with research limited to salamanders [17–20], fishes [20–22], amphipods [16], and crayfishes [21, 23]. Korbel et al. [24] applied a metabarcoding approach to characterize the DNA community of prokaryotes and eukaryotes using 16S rDNA and 18S rDNA, respectively. These studies have demonstrated the utility of eDNA for detecting rare and threatened groundwater biodiversity as an effective complement and possible alternative to more invasive and destructive traditional sampling approaches.

**Fig 1:**
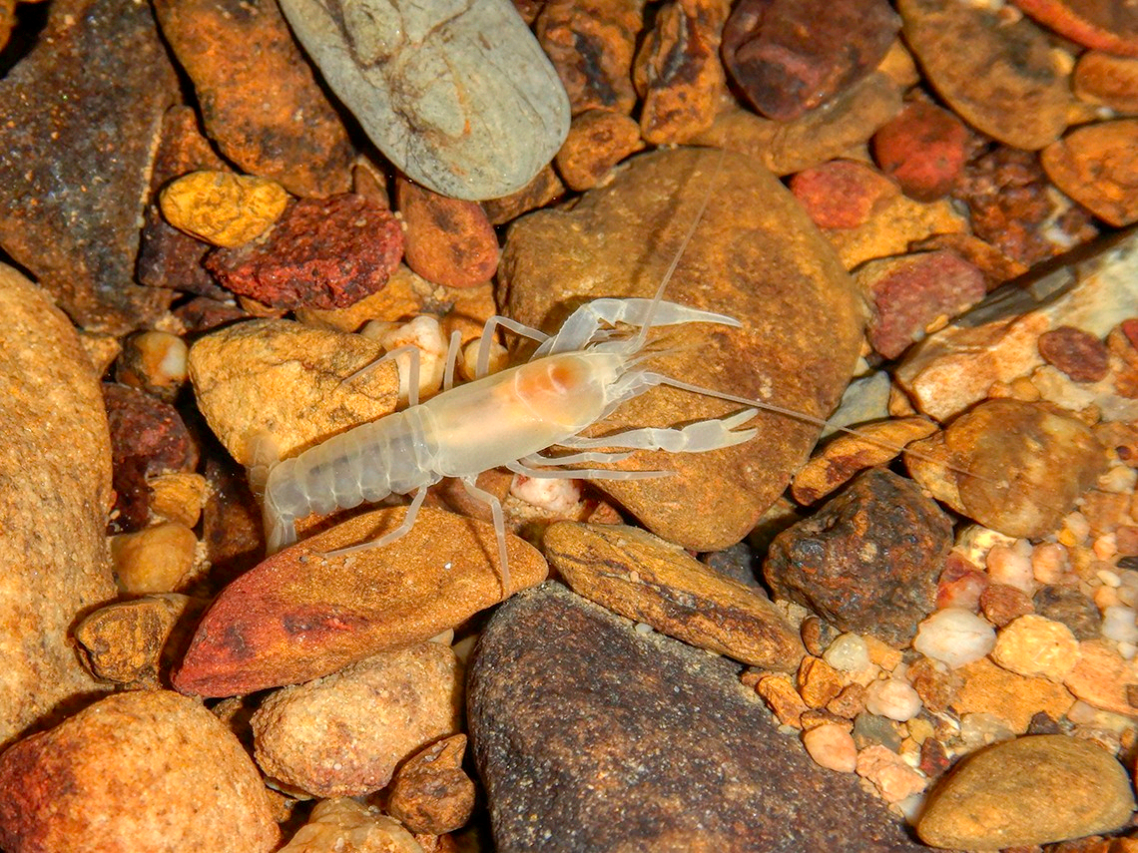
The Sweet Home Alabama Cave Crayfish (*Cambarus speleocoopi*) is an obligate groundwater crayfish endemic to just four cave systems in Marshall Co., Alabama, USA. Photo by Matthew L. Niemiller.

The Sweet Home Alabama Cave Crayfish, *Cambarus speleocoopi*, is a blind, depigmented cave-dwelling crayfish (Fig 1) in the subgenus *Aviticambarus* recently described by Buhay and Crandall [25] that is closely related to several other groundwater crayfishes in northern Alabama, including *C*. *hamulatus*, *C*. *jonesi*, and *C*. *laconensis*. This obligate cave-dweller is endemic to Marshall County, Alabama, occurring in just four cave systems along both sides of the Tennessee River valley northwest and downstream of Guntersville Dam [25, 26]. Cave bioinventory surveys have not yielded any additional occurrences in recent years. Because of its extremely limited distribution (extent of occurrence 61.5 km^2^) and presumed rarity, *C*. *speleocoopi* is considered a priority species of high conservation concern (Priority 2) in Alabama [26]. Its conservation status has been evaluated as Critically Imperiled (G1) by NatureServe [27] and Endangered under criteria B1ab(v) by IUCN [28]. Potential threats to the species include groundwater pollution associated with urban development and changes to hydrology related to impoundments on the Tennessee River [25, 28].

Few cave systems in the Tennessee-Alabama-Georgia (TAG) region have been comprehensively surveyed biologically [29, 30], and there is great potential that species such as *C*. *speleocoopi* occur at additional undocumented sites. In fact, cavers regularly report observations of “white and blind crayfishes” from undocumented cave systems in northern Alabama. However, species identification requires specimen collection and genetic analysis, as morphology alone cannot easily distinguish *C*. *speleocoopi*, *C*. *laconensis*, and *C*. *jonesi* [26]. In fact, populations of *C*. *speleocoopi* from Beech Spring and Keller’s caves in Marshall County were identified as *C*. *jonesi* previously [31]. Consequently, eDNA may be an appealing, nondestructive alternative to rapidly determine species occupancy and identification at a spring, cave, or well for rare, threatened, or endangered groundwater species without the need for specimen collection and expert identification.

In this study, we developed, tested, and validated an eDNA assay for *C*. *speleocoopi* and screened water samples collected from springs and cave systems (including historical sites) within and near its distribution to test the applicability of an eDNA approach to detect a karst groundwater crustacean and identify possible new sites of this imperiled crayfish. Our study demonstrates the potential utility of eDNA as an effective surveying and monitoring tool for groundwater biodiversity but also highlights some challenges of this approach when applied to groundwater ecosystems.

## Methods

### Ethics statement

This research was authorized under Alabama Department of Conservation and Natural Resources scientific collection permit nos. 2018061776268680 and 2019060224868680 and Alabama State Parks scientific permit no. 192213. Cave location data has been intentionally omitted to protect these sensitive ecosystems and their biodiversity. Cave location data are maintained by the Alabama Cave Survey (ACS; http://www.alabamacavesurvey.org) and can be requested from ACS or the corresponding author.

### Sampling sites

**Fig 2:**
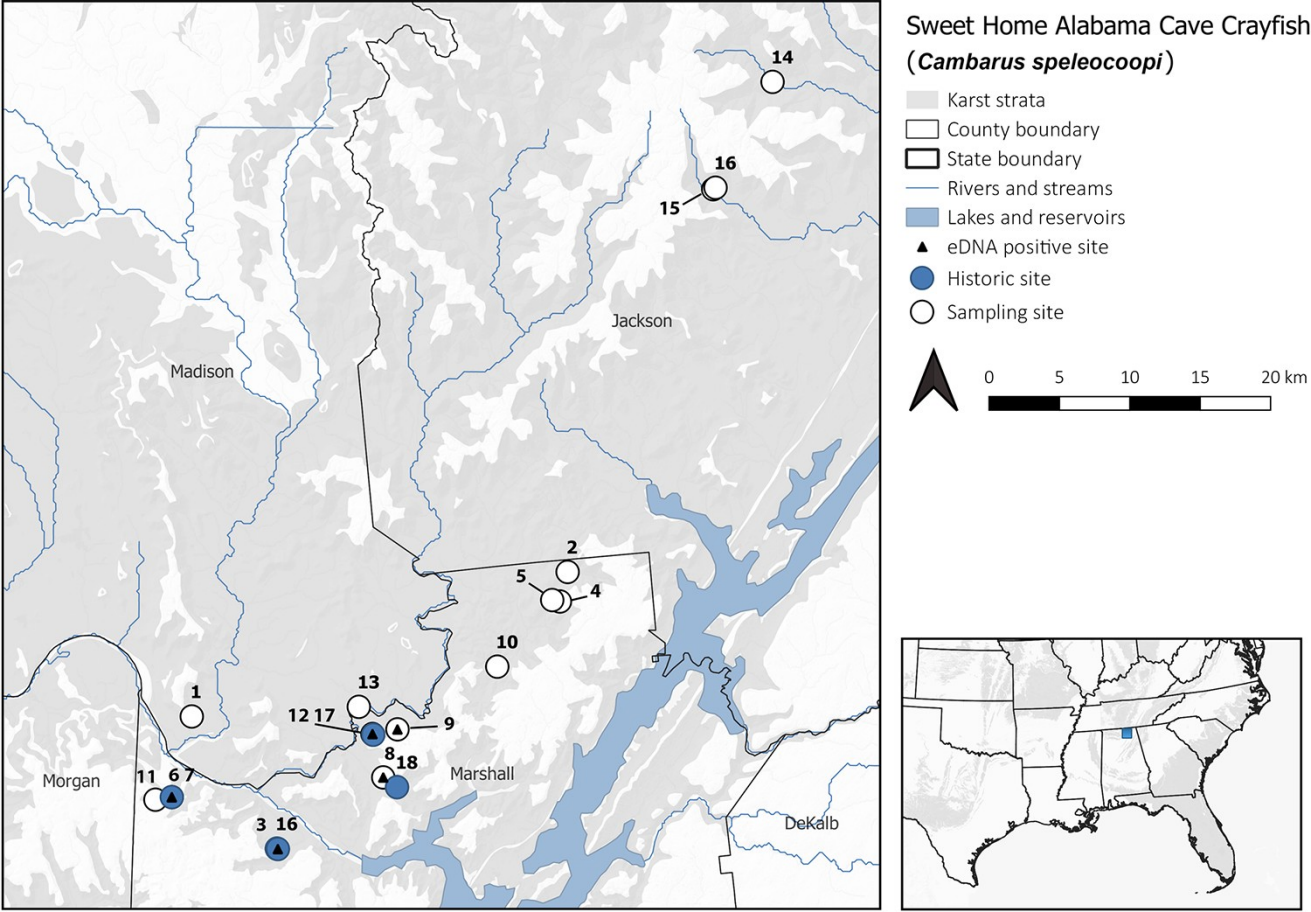
Distribution of the Sweet Home Alabama Cave Crayfish (*Cambarus speleocoopi*) (blue dots) and eDNA sampling sites (white dots and numbered blue dots) in northern Alabama, USA. *Cambarus speleocoopi* eDNA was detected (black triangle) at two historical sites and three new sites. Site numbers correspond to those listed in Table 1. Sites 17 and 18 are historical sites but we could not gain permission to sample. Karst and cave-bearing carbonate strata is shown in gray.

We collected water samples from 13 sites between March 2018 and April 2019 (Table 1), including two historical sites–Cherry Hollow Cave (and the associated spring run) and Beech Spring Cave. *Cambarus speleocoopi* has been confirmed at these sites within the past three years (Niemiller, unpublished data). We could not obtain permission to access Keller’s Cave (site 17) (but collected water at the nearby spring; site 12) and Porches Spring Cave (site 18). We also sampled 10 additional sites (three caves and seven springs) located within a 20-km radius of historical sites in Jackson and Marshall counties, Alabama (Table 1; Fig 2). Three sites (sites 14–16 in Table 1) outside of the suspected range of *C*. *speleocoopi* were included to serve as negative field controls.

**Table 1:** Sampling sites where water samples were collected in Jackson and Marshall counties, Alabama, USA and results of screening of an eDNA assay for *Cambarus speleocoopi*.

Site no.	County	Site name	Collection date	Technical replicates
1	Marshall	Ashburn Spring	11 Jan 2019	0/12
2	Marshall	Babe Wright Spring	07 Feb 2019	0/24
3	Marshall	Beech Spring Cave (spring)^a^	29 Apr 2019	6/12
4	Marshall	Cathedral Caverns	07 Feb 2019	0/12
5	Marshall	Cathedral Caverns (spring)	07 Feb 2019	0/12
6	Marshall	Cherry Hollow Cave^a^	18 Jan 2019	5/12
7	Marshall	Cherry Hollow Cave (spring)^a^	18 Jan 2019	3/12
8	Marshall	Cushion Spring	11 Jan 2019	2/12
9	Marshall	Davis Spring	11 Jan 2019	1/18
10	Marshall	Guffey Cave	22 Feb 2019	0/12
11	Marshall	Kings Spring Cave	01 Feb 2019	0/18
12	Marshall	McGehee Spring	11 Jan 2019	1/12
13	Marshall	New Hope Spring	31 Jan 2019	0/12
14	Jackson	Bluff River Cave^b^	18 Aug 2018	0/12
15	Jackson	Tumbling Rock Cave^b^	04 Mar 2018	0/12
16	Jackson	Tumbling Rock Cave (spring)^b^	04 Mar 2018	0/12
17	Marshall	Keller’s Cave ^a^		
18	Marshall	Porches Spring Cave ^a^		

^a^Historical sites for *C*. *speleocoopi*.

^b^Sites used for negative field controls. Two to four water samples were collected from a site, each with six PCR technical replicates. Sites 17 and 18 are historical sites but we could not gain permission to sample.

### Water sampling, filtering, and eDNA filter extraction

We collected 1–2 L of water in total (2–4 500 mL samples) at each site by submerging sterile Nalgene bottles beneath the surface. For spring sites, we collected water samples as close as possible to the point where water emerged from underground. Water samples were collected, placed on ice in a cooler, then transported back to the laboratory at The University of Alabama in Huntsville for filtering.

Water samples were vacuum-filtered within 24 hours of collection in the laboratory using 0.45-μm cellulose-nitrate filters (Thermo Scientific^TM^ Nalgene^TM^) following Niemiller et al. [16]. After filtration, the filters were folded, transferred to 8-mL sterile tubes, then stored at -20°C until DNA extraction. Multiple filters were needed for a few water samples with substantial amounts of suspended silt and organic matter. For each set of water samples filtered, we also filtered a water sample comprised of distilled water to serve as a negative control.

We extracted DNA from filters in a dedicated laminar flow hood using a modified Qiagen® DNeasy Blood and Tissue Kit (Qiagen Inc., Valencia, CA, USA) protocol. One half of each filter was cut into small pieces using flame-sterilized scissors and transferred into a 2-mL microcentrifuge tube. The remaining half of each filter was stored long-term to serve as a back-up. For environmental water samples that required multiple filters, we extracted DNA from one-half of each filter and then pooled elutions. We added 360 μL ATL Buffer and 40 μL of Proteinase K (Qiagen, Inc.) then incubated filter pieces overnight at 55°C. Samples were then vortexed for 15 s and centrifuged for 1 min (8000 g). The resulting supernatant was transferred into a new 2-mL microcentrifuge and processed following the manufacturer’s protocol, with the exceptions of using 400 μL of AL Buffer and 400 μL of ethanol. The final elution volume for all samples was 125 μL of buffer AE preheated to 70°C. In addition, elutions were treated with the OneStep™ PCR Inhibitor Removal Kit (Zymo Research) to remove potential PCR inhibitors that may be present. Elutions were stored at -20°C until qPCR.

### qPCR assay design

Candidate species-specific qPCR assays that included forward and reverse primers and an intervening hydrolysis probe were designed using Integrated DNA Technologies’(IDT) PrimerQuest tool [32] available online at https://www.idtdna.com/PrimerQuest/Home/Index. The default settings for qPCR assay design were used except for modifying the optimal primer temperature (60°C), optimal probe temperature (70°C), and amplicon length (200 bp max). CO1 sequences available on GenBank for *C*. *speleocoopi* (accession nos. DQ411780–DQ411781; [33]) as well as other crayfish species that may occur in the study area were aligned using MUSCLE [34] in the program Jalview [35]. A consensus sequence from this alignment for *C*. *speleocoopi* was used as input into PrimerQuest.

### *In silico* and *in vitro* assay validation

We examined specificity of candidate assays both *in silico* and *in vitro*. For *in silico* validation, we used NCBI Primer-BLAST to query forward and reverse primers and probes against the nr/nt database [36]. Candidate assays then were synthesized by IDT; the internal probe was a PrimeTime® double-quenched ZEN™/IOWA Black™ FQ probe labeled with 6-FAM and validated *in vitro*. We tested specificity *in vitro* by qPCR on a 678-bp gBlock gene fragment synthesized by IDT based on GenBank accession no. DQ411780 for *C*. *speleocoopi* and tissue-derived genomic DNA for *C*. *speleocoopi* and other crayfish species in the family Cambaridae, that may potentially occur in caves and springs in the study area, including *C*. *hamulatus*, *C*. *jonesi*, *C*. *laconensis*, *C*. *pecki*, *C*. *tenebrosus*, and *Orconectes australis*. We evaluated performance of our candidate assays after optimizing primer-probe concentrations and qPCR reagents and settings for our field samples (see below).

### Primer-probe concentration optimization

We optimized concentrations of primers and probe by screening varying concentrations of forward and reverse primers (600 nM, 900 nM, and 1200 nM) and probe (125 nM, 250 nM, and 500 nM) in triplicate with 0.5 ng of target-species synthetic DNA per 20- μL reaction (see next section). Primer-probe concentrations with the greatest peak fluorescence and lower Ct values were employed in screening field-collected water samples.

### Field eDNA sample screening

Real-time qPCR amplification of each field-collected water sample was conducted with six replicates in a final volume of 20 μL, using 10.0 μL of TaqMan® Environmental Master Mix 2.0 (Applied Biosystems), 4.7 μL of ddH20, 0.9 μL of forward primer (20 μM), 0.9 μL of reverse primer (20 μM), 0.5 μL of probe (20 μM), and 3.0 μL of template DNA. Final concentrations of primers and probe in each 20-μL reaction were 900 and 250 nM, respectively. Samples were run in 96-well optical plates on a QuantStudio® 3 Real-Time PCR System (Applied Biosystems) under the following conditions: an initial 10-min incubation at 95°C to activate the AmpliTaq Gold® enzyme followed by 50 cycles of denaturation at 95°C for 15 s and annealing/extension at 60°C for 1 min. A dilution series of *C*. *speleocoopi* synthesized gBlock was used as a positive control standard, ranging from 10^−1^ to 10^−9^ ng/μL in concentration. This standard also was used to determine the limit of detection (LOD)–the concentration in which there was at least one positive amplification among technical replicates, and limit of quantification (LOQ)–the concentration in which all technical replicates amplified. Negative controls with all PCR reagents but no template (six replicates) were included on each plate to assess potential contamination. We also screened negative filter controls. Positive amplifications were purified using ExoSAP-IT and specificity confirmed via Sanger sequencing at Eurofins Genomics (Louisville, Kentucky, USA).

### Contamination precautions

False positives can result from contamination during every step in the sampling and quantification pipeline from field collection and filtering of water samples to DNA extraction and qPCR amplification. To minimize the potential for contamination, we employed several procedures in addition to those already outlined. Prior to water sample collection in the field, all bottles and collection supplies were sterilized with 10% bleach solution and autoclaved. Filtering occurred in a lab space dedicated for such purposes. All eDNA filter extractions and qPCR preparations were conducted in dedicated laminar flow hoods. Lab space surfaces and equipment (e.g., pipettes, forceps, tubes, and other consumables etc.) were decontaminated before and after use with 10% bleach solution and/or 30-min ultraviolet light (UV) treatment. Filtered tips were used during all protocols that required pipetting. Finally, disposable gloves were worn during field collection, filtering, DNA extraction, and qPCR setup and cleanup. We included negative controls during the filtering, DNA extracting, and qPCR amplification stages to check for potential contamination.

## Results

### Assay design and validation

The species-specific qPCR assay developed for *C*. *speleocoopi* targeted a 163-bp fragment of the mitochondrial CO1 locus (Table 2). In silico assay validation demonstrated that this assay was not likely to amplify non-target crayfish species, particularly other cave-dwelling crayfishes in the genus *Cambarus* that also occur in the Tennessee River Valley of northern Alabama–*C*. *hamulatus*, *C*. *jonesi*, *C*. *laconensis*, *C*. *pecki*, and *C*. *tenebrosus*. The forward primer had one mismatch with published sequences of *C*. *jonesi* (GenBank accession nos. DQ411777–DQ411779), two mismatches with *C*. *laconensis* (accession no. DQ411782) and *C*. *pecki* (accession no. JX514434), 1–2 mismatches with *C*. *hamulatus* (accession nos. DQ411760–DQ411776) and *C*. *tenebrosus* (accession no. EU583576 and JX514444), and 1–3 mismatches with *O*. *australis* (accession nos. EF207161–EF207162, EU583506–EU583551, EU583577–EU583580, EU583583–EU583604, EU583607–EU583620, EU583622–EU583626, EU583628). The reverse primer had 2–3 mismatches with published sequences of *C*. *hamulatus*, *C*. *laconensis*, *C*. *pecki*, *C*. *tenebrosus*, and *O*. *australis*, and one mismatch with published sequences of *C*. *jonesi*. Finally, the probe had 4–5 mismatches with published sequences of *C*. *hamulatus*, *C*. *jonesi*, *C*. *laconensis*, *C*. *pecki*, and *C*. *tenebrosus*, and 3–5 mismatches with published sequences of *O*. *australis*. In vitro validation also showed that the designed assay was specific to *C*. *speleocoopi* with a LOD of 1–2 copies/μL and LOQ of 13.5 copies/ μL (R^2^ = 0.995).

**Table 2:** Primers and probe developed and used in the current study to amplify a 163-bp fragment of CO1 for *Cambarus speleocoopi*.

Oligo	Sequence (5’ to 3’)	Direction	Length (bp)	Tm (°C)
Forward	TGGGATAGTTGGGACTTCA	Sense	19	60
Reverse	ATTRCCAAACCCTCCAATTA	Antisense	20	60
Probe	TCCGAGTTGAATTGGGTCAGGTAGGAAGG	Sense	29	70

### Field surveys

We detected *C*. *speleocoopi* eDNA at 5 of 16 sites sampled, including two historical sites–Beech Spring Cave at the spring (site 3) and Cherry Hollow Cave (both in the cave and at the spring; sites 6 and 7). We also detected *C*. *speleocoopi* at three additional and potentially new sites in Marshall County–Cushion Spring (site 8), McGehee Spring (site 12), and Davis Spring (site 9). All three of these sites were within 2 km of historical sites for the species. Positive detections from Cherry Hollow Cave (in cave and at the spring) were identical to the corresponding region of a CO1 haplotype from the cave sampled previously (accession no. D411780). Positive detections from Beech Spring Cave, Cushion Spring, McGehee Spring, and Davis Spring matched a CO1 haplotype from Keller’s and Porches Spring Cave (accession no. D411781). We did not detect *C*. *speleocoopi* eDNA at Bluff River Cave (site 14) and Tumbling Rock Cave (site 15) and associated spring (site 16) in Jackson County. These sites are far removed from the range of *C*. *speleocoopi*, but populations of the closely related *C*. *hamulatus* occur in these two caves. We found no evidence of contamination in either our field or laboratory controls across qPCR runs. All positive detections were confirmed by Sanger sequencing.

## Discussion

The use of eDNA as an effective approach in the detection and monitoring of groundwater fauna is still in its infancy. However, our study contributes to a growing literature that indicates eDNA is a viable monitoring tool for occupancy of a fauna that is otherwise difficult to survey and study using traditional approaches. To date, single-species eDNA assay approaches have been applied successfully in the detection of two groundwater salamanders—Olm (*Proteus anguinus*) from caves, springs, and wells in Bosnia and Herzegovina, Croatia, Montenegro, and Slovenia [17–19] and Barton Springs Salamander (*Eurcyea sosorum*) from a spring in the Edwards Aquifer region of Texas, USA [20], four groundwater fishes—Mexican Blindcat (*Prietella phreatophila*) from a cave in the Edwards Aquifer region of Texas, USA [20], Blind Cave Eel (*Ophisternon candidum*) from boreholes in northwestern Australia [22], and Ozark Cavefish (*Troglichthys rosae*) and Eigenmann’s Cavefish (*Typhlichthys eigenmanni*) from caves, springs, and wells in the Ozarks region of Arkansas, Missouri, and Oklahoma, USA [21], two amphipods—Hay’s Spring Amphipod (*Stygobromus hayi*) and Potomac Groundwater Amphipod (*S*. *tenuis potomacus*) from hypotelminorheic springs in the District of Columbia, USA [16], and two crayfishes—Oklahoma Cave Crayfish (*Cambarus tartarus*) from caves in the Ozarks region of Oklahoma, USA [21] and Caney Mountain Cave Crayfish (*Oroconectes stygocaneyi*) from a cave in the Ozarks region of Missouri, USA [21, 23]. This study is the first to successfully detect eDNA of crayfishes from groundwater of the Interior Plateau karst region and demonstrates that eDNA can detect groundwater crustaceans in karst groundwater habitats, such as springs and cave streams.

We designed a species-specific assay that was lab and field validated to discriminate *C*. *speleocoopi* from several closely related, morphologically cryptic congeners. Our assay detected *C*. *speleocoopi* at two historical sites where occupancy has been confirmed by visual surveys within the last five years. However, we also detected *C*. *speleocoopi* eDNA at three potentially new sites in Marshall County, suggesting that an eDNA approach is a rapid and cost-effective method to detect rare subterranean fauna of conservation concern inhabiting karst groundwater. The three positive detections of *C*. *speleocoopi* eDNA from Cushion, Davis, and McGehee springs (sites 8, 9, and 12, respectively) appear reasonable given the close proximity to historical sites north of the Tennessee River on the escarpments of Grassy Mountain (Fig 2). Cushion Spring is located 1.2 km northwest of Porches Spring Cave (site 18) on the south side of Grassy Mountain. McGehee Spring is located just 55 m north and is the main resurgence of the stream in Keller’s Cave (site 17), which is the type locality of *C*. *speleocoopi* located on the north side of Grassy Mountain. Davis Spring is located 1.9 km east of Keller’s Cave. All of these springs and caves drain into the Paint Rock River. However, eDNA samples from Kings Spring Cave (site 11) located on the south side of Kings Hollow on Brindley Mountain in the Little Cane Creek drainage south of the Tennessee River did not detect *C*. *speleocoopi* eDNA, despite being just 1.2 km west of Cherry Hollow Cave (site 6). *Cambarus speleocoopi* has never been observed at Kings Spring Cave, including several recent surveys since 2018. Hydrological connectivity between these cave and spring sites is not well understood, as dye tracing investigations have yet to be conducted for these particular karst cave systems.

It is possible that *C*. *speleocoopi* is present but rare (i.e., in low abundance) at non-detection sites in Marshall County, but our assay was not sensitive enough to detect extremely low abundance. Conducting eDNA studies on groundwater crayfishes (and other groundwater fauna) in an occupancy-modelling framework [37, 38] would benefit future research efforts [e.g., 19]. In addition, we did not assess the effects of water level/flow on detectability. Water samples were collected primarily in late winter and early spring when local water tables were higher, as some spring sites have little to no flow in summer and autumn. However, we only sampled when water conditions were considered normal for the time of the year, and avoided collecting water samples after recent heavy rainfall events that resulted in higher water levels in caves, increased discharge from karst springs, and increased sediment transport, as water volume, flow, and sediment load all can influence eDNA detection [39–42]. Regardless, eDNA concentrations may vary seasonally in caves and springs due to variation in environmental factors, as observed in other aquatic systems [e.g., 43] and suggested for cave systems [21], such as water volume, flow rate, concentrations of potential inhibitors, etc., as well as biological factors, such as population dynamics and seasonality of reproduction. For example, seasonal variation in crayfish life history and behavior may influence detectability using eDNA [44]; however, little is known about the life history of *C*. *speleocoopi*. These and other factors can affect the production rate of eDNA. We encourage future researchers to examine seasonal and annual variation in eDNA concentration and detectability.

Several abiotic and biotic factors affect the degradation and persistence time of eDNA in water, such as UV radiation, temperature, pH, and microbial activity [45–52]. A wide range of degradation rates of eDNA have been reported in the literature for aquatic species in surface ecosystems [12, 48]; however, estimates from groundwater habitats are unknown. In general, degradation rates are greater at warmer temperatures [46, 48], higher UV radiation exposure [45, 46, 50, but see 51], and higher levels of microbial activity [45, 46, 49, 52]. Groundwater ecosystems are characterized by lack of light (and UV radiation), cooler and more stable temperatures, lower nutritional resources, and lower microbial activity compared to most surface aquatic ecosystems [53, 54]. Consequently, eDNA is thought to be comparatively stable in groundwater habitats and capable of being detected [18]. However, the persistence time of eDNA in groundwater is unknown but likely is considerably longer than the timescale of hours to days reported for many surface species and habitats. Macro-organismal eDNA has been recovered from dry cave sediments dating back thousands to hundreds of thousands of years before present [e.g., 55–57], demonstrating the potential for subterranean habitats to act as incubators for eDNA.

Determining degradation rates and persistence time of eDNA in various groundwater habitats should be priority of future research, as inaccurate estimates may have implications for conservation and management. Many groundwater and cave-dwelling species have small, restricted distributions (i.e., short-range endemics) and are of conservation concern [58, 59]. An underlying assumption of eDNA approaches for detection and monitoring is that a positive detection represents contemporary occupancy. However, if eDNA persists for weeks to months or even years in groundwater habitats, then we could overestimate the distribution of a species–e.g., a “false positive” in which eDNA is detected at a site where a population has long been extirpated–or fail to detect declines in range size when eDNA is employed as the sole approach in a monitoring program.

The use of eDNA in the detection and monitoring of groundwater fauna is still in its early stages. Here we report the first application of eDNA to detect a stygobitic crayfish in karst groundwater habitats of the Interior Plateau karst region and provide a demonstration for how eDNA can be applied in groundwater biodiversity monitoring of rare and endangered taxa. Our study along with the encouraging results from the few other recent studies to date have shown that eDNA can be a valid and effective complement to traditional sampling approaches for determining occupancy of groundwater species. We envision a quick transition from proof-of-concept studies to experimental approaches examining rates of degradation and persistence in groundwater to development of best practices for long-term monitoring programs of groundwater fauna.
